# Frequency and Determinants of Levothyroxine Therapy Initiation for Veterans with Subclinical Hypothyroidism

**DOI:** 10.3390/jcm13195727

**Published:** 2024-09-26

**Authors:** Andreea Terlea, Freddy J. K. Toloza, Richard R. Owen, James S. Williams, Micheal Knox, Katherine Dishongh, Jeff D. Thostenson, Naykky M. Singh Ospina, Juan P. Brito, Spyridoula Maraka

**Affiliations:** 1 Central Arkansas Veterans Healthcare System, Little Rock, AR 72205, USA; acterlea@uams.edu (A.T.); richard.owen2@va.gov (R.R.O.); james.williams17745e@va.gov (J.S.W.); micheal.knox@va.gov (M.K.); katherine.dishongh@va.gov (K.D.); 2 Division of Endocrinology and Metabolism, Department of Internal Medicine, University of Arkansas for Medical Sciences, Little Rock, AR 72205, USA; freddy.tolozabonilla@nih.gov; 3 Diabetes and Endocrinology and Obesity Branch, National Institute of Diabetes and Digestive and Kidney Disorders, National Institutes of Health, Bethesda, MD 20892, USA; 4 Department of Psychiatry, University of Arkansas for Medical Sciences, Little Rock, AR 72205, USA; 5 Department of Biostatistics, University of Arkansas for Medical Sciences, Little Rock, AR 72205, USA; jdthostenson@uams.edu; 6 Division of Endocrinology, Department of Medicine, University of Florida, Gainesville, FL 32610, USA; naykky.singhospina@medicine.ufl.edu; 7 Division of Endocrinology, Diabetes, and Metabolism, Department of Medicine, Mayo Clinic, Rochester, MN 55905, USA; brito.juan@mayo.edu

**Keywords:** thyroid, levothyroxine, hormone replacement, subclinical hypothyroidism, autoimmunity, treatment

## Abstract

**Background/Objectives:** There is evidence of overtreatment in patients with subclinical hypothyroidism (SCH). We aimed to identify the proportion of patients treated for SCH and the determinants of thyroid hormone therapy initiation. **Methods:** We included a random sample of adult Veterans diagnosed with SCH from 1 January 2016 to 31 December 2018 and conducted univariate and multivariable logistic regression to identify factors associated with levothyroxine initiation. **Results:** Out of 229 Veterans with SCH [90.0% male, 87.2% White, 99.1% non-Hispanic, median age (interquartile range; IQR) 68 (17) years], 27.5% were treated with levothyroxine. The treated group had a higher proportion of White patients (95.2% vs. 84.2%, *p* = 0.039), a higher thyrotropin level [median (IQR), 6.98 (2.06) mIU/L vs. 6.14 (1.10) mIU/L, *p* = 0.0002], a higher proportion of patients with thyrotropin level ≥ 10 mIU/L (11.1% vs. 3.0%, *p* = 0.021), a lower frequency of confirmatory thyroid testing before initiating levothyroxine (49.2% vs. 97.0%, *p* < 0.0001), and a similar frequency of thyroid autoimmunity testing (3.2% vs. 0.6%, *p* = 0.18) compared to the untreated group. In a multivariable logistic regression analysis, White race (OR = 4.50, 95% CI 1.19 to 17.08, *p* = 0.026) and index thyrotropin level [OR = 1.71, 95% CI 1.24 to 2.35, *p* = 0.001; for every SD increase (1.6 mIU/L)] were associated with higher odds of treatment. **Conclusions:** Three in 10 Veterans with SCH received levothyroxine, often based on a single abnormal thyroid test without autoimmunity assessment. White race and higher thyrotropin level were linked to increased odds of starting treatment, indicating potential disparities and the influence of SCH severity on decision-making.

## 1. Introduction

Subclinical hypothyroidism (SCH) is an increasingly frequent biochemical condition diagnosed when a patient presents with mildly high thyrotropin (TSH) levels yet normal free thyroxine (FT4) levels. It affects up to 20% of adults, of whom 70% will have nonspecific symptoms and the rest will have no symptoms at all [1,2]. In contrast, overt hypothyroidism (OH), diagnosed by elevated TSH and low FT4 levels, is a typically symptomatic, less common condition, with a stable incidence of 3 in 1000 adults [3]. Current guidelines recommend treating all patients with OH with levothyroxine (LT4), a synthetic form of the thyroid hormone thyroxine, due to the proven benefit of treatment [4,5].

According to the National Health and Nutrition Examination Survey III [3], approximately 14 million people in the United States have SCH. The condition is more common in women and in White individuals, and it increases with age [1,3]. However, TSH levels also increase with age [6]; therefore, a small elevation in TSH levels may be normal for older adults. In addition, approximately 9 in 10 patients who are diagnosed with SCH have a TSH level between 4 and 10 mIU/L [7]. However, in 62% of individuals with an initial TSH level of 5.5–10 mIU/L, the TSH level normalizes without any intervention in a subsequent measurement [8], which has been attributed to biological variation in TSH levels, drugs, concomitant transient disease, and temporary increase in response to environmental and stress factors [9]. Therefore, guidelines recommend confirming the diagnosis of SCH before initiating LT4 therapy by repeating thyroid function tests (TFTs) [4,10]. Moreover, guidelines recommend LT4 treatment for some patients with SCH based on their symptoms, age, presence of thyroid autoimmunity, cardiovascular risk factors, and pregnancy considerations [5,10]. Conversely, a guideline panel [11] recommended against thyroid hormone treatment for most adults with SCH based on the lack of benefits in randomized clinical trials [12].

There is substantial evidence for the overtreatment of patients with SCH [13,14,15], exposing them to unnecessary burden and risk of harm [16,17,18,19,20]. It is also important to recognize that disparities in LT4 initiation have been identified in the context of increasing LT4 prescriptions in the general population and across sociodemographic groups. Understanding the drivers of LT4 therapy initiation among patients with SCH necessitates examining the frequency and associated factors of LT4 prescriptions in this population. To achieve this, we performed an observational cohort study to determine the proportion of Veterans with SCH being initiated on thyroid hormone therapy and to identify the determinants influencing decisions regarding thyroid hormone therapy initiation for SCH.

## 2. Materials and Methods

### 2.1. Study Design

In this retrospective cohort study, using the Corporate Data Warehouse through the VA Informatics and Computing Infrastructure, we initially identified 1023 Veterans aged 18–89 years old (at the time of index TSH) who had at least one elevated TSH level (index TSH) from 1 January 2016 to 31 December 2018 at the Central Arkansas Veterans Healthcare System (CAVHS), Little Rock, AR, USA, and no history of abnormal FT4 within 3 months from the index TSH, use of thyroid-affecting medications the last 12 months from index TSH, thyroidectomy, or neck radiation.

We then selected a random sample of 450 patients using SAS 9.4 to screen for study eligibility based on an electronic health record (EHR) review. We included Veterans aged 18–89 years old (at the time of index TSH) who met the laboratory criteria for SCH [at least one set of elevated TSH and normal FT4 or total thyroxine (TT4) levels during the study period, the first instance of elevated TSH was considered “index TSH” and used for analysis] from 1 January 2016 to 31 December 2018 at CAVHS. SCH was diagnosed based on the CAVHS laboratories’ reference range for TSH and FT4/TT4 at the time of testing. We excluded Veterans who (1) did not have a follow-up appointment with either primary care or the specialty who ordered the index TSH within one year, (2) were treated with thyroid hormones or antithyroid drugs (methimazole, propylthiouracil) prior to their index TSH test, (3) were pregnant or delivered within 6 months of their index TSH test, (4) were critically ill or had a recent hospital stay (4 weeks prior to index TSH), (5) were on weight loss (naltrexone-bupropion, orlistat, phentermine, or phentermine-topiramate) or thyroid-affecting medications (lithium, amiodarone, or tyrosine kinase inhibitors), and (6) had a history of OH, hyperthyroidism, thyroidectomy, radioactive iodine therapy, or neck radiation.

### 2.2. Data Collection

The EHR review aimed to identify the factors that may influence the decision to initiate LT4. We collected data using a standardized extraction form in a secure web application designed for research data collection (REDCap), which is compliant with HIPAA regulations. Quality control measures were implemented to verify the accuracy and plausibility of the data, including data validation checks, data consistency checks, and random sample audits. We extracted patients’ demographic and social characteristics (sex, race, ethnicity, marital status, employment status), hypothyroidism-related symptoms (e.g., weight gain, fatigue, constipation), comorbidities (e.g., goiter, cardiovascular disease, hyperlipidemia), smoking status, family history of thyroid disorders, vitals (weight, height, body mass index, blood pressure, heart rate), physical exam findings, TFTs with dates and values, and thyroid autoimmunity status (thyroid peroxidase antibody or thyroglobulin antibody positivity). Data related to thyroid hormone treatment (e.g., confirmation of SCH with a second set of TFTs before receiving LT4 prescription), justification for thyroid hormone prescription, the type and initial dose of thyroid hormone replacement therapy, and clinician characteristics (medical specialty, clinician type) were also extracted.

### 2.3. Statistical Analysis

We performed a descriptive summary analysis of the patients’ baseline characteristics grouped by LT4 treatment status. Data are presented as frequencies (percentages) for categorical variables and means (standard deviation; SD) or medians (interquartile range; IQR) for continuous variables based on data normality. Differences between categorical variables were assessed using Fisher’s exact test, and differences between continuous variables were assessed using Welch’s two-sample *t*-test, *t*-test for unequal variances, or Wilcoxon Rank-Sum test based on data distribution.

The primary outcome was to determine the proportion of Veterans with SCH being initiated on LT4 therapy. The secondary outcome was to identify the determinants of LT4 prescription for SCH. For the primary outcome, we calculated the proportion of patients with SCH who were prescribed LT4 therapy. Assuming that the LT4 initiation rate would be less than 30%, we needed to identify at least 202 eligible patients to attain a 5% margin of error for our estimate. For the secondary outcome, multivariable logistic regression analysis was performed with the most clinically relevant variables and those that were significantly different among the treatment groups (age, sex, race, presence of any thyroid symptoms, and index TSH level). The results of the multivariable logistic regression analysis are reported as odds ratios (OR) and 95% confidence intervals (CI). We also aimed to examine the factors that may be associated with the initial LT4 dose by fitting univariate and multivariable linear regression models. A *p* < 0.05 was considered to be statistically significant, and all testing were 2-sided. All statistical analyses were performed using R 4.3.2.

## 3. Results

### 3.1. Sociodemographic, Clinical and Treatment Characteristics

Two hundred and twenty-nine patients with SCH were identified after the EHR review. Of these, 90.0% were male, 87.2% were White, 99.1% were non-Hispanic, and the population median age (IQR) was 68 (17) years. Sixty-three patients (27.5%) diagnosed with SCH were initiated on thyroid hormone treatment. LT4 prescription was used in all cases, with starting LT4 dose ranging from 25 to 88 µg /day (25th–75th percentile = 25–50 µg daily).

Table 1 includes a summary of the demographic and social characteristics of each treatment group. The treated group had a higher proportion of White patients (95.2% vs. 84.2%, *p* = 0.039) compared to the untreated group.

No significant differences were found in hypothyroidism-related symptoms (Table 2) and comorbidities (Table 3) between the treated and untreated groups. Additional clinical characteristics of the treatment groups are displayed in Table 4.

### 3.2. Biochemical Data

The treated group had a higher index TSH level [median (IQR), 6.98 (2.06) mIU/L vs. 6.14 (1.10) mIU/L, *p* = 0.0002] and a higher proportion of patients with TSH level ≥ 10 mIU/L (11.1% vs. 3.0%, *p* = 0.021) compared to those in the untreated group. There was no significant difference between the treated and untreated groups in FT4 levels [mean (SD), 0.79 (0.15) ng/dL vs. 0.82 (0.12) ng/dL, *p* = 0.23]. There was no significant difference in the frequency that thyroid autoimmunity was assessed (3.2% vs. 0.6%, *p* = 0.18); however, only 1.3% of the patients underwent thyroid autoimmunity labs.

Only 49.2% (31/63) of the patients in the treated group, compared to 97.0% (161/166) of the patients in the untreated group, had confirmatory TFTs before the decision to initiate LT4 therapy was made (*p* < 0.0001). When repeat TFTs were obtained, TSH levels normalized in 71.9% (138/192) of the patients. Of the patients with repeat TFTs, SCH was confirmed in 96.8% (30/31) of the treated patients and 14.9% (24/161) of the untreated patients. Table 4 includes a summary of the biochemical data for each treatment group.

### 3.3. Clinician Characteristics

Index TSH was predominantly ordered by primary care clinicians (94.3%), who also provided the majority of LT4 prescriptions (96.8%). More patients whose index TSH testing was ordered by a physician’s assistant/nurse practitioner were subsequently treated compared to those whose index TSH testing was ordered by physicians [35.4% (29/82) vs. 23.1% (34/147), *p* = 0.06]. The primary justification documented in the clinical notes for prescribing LT4 therapy was abnormal TFTs (92.1%), followed by presenting symptoms at diagnosis (4.8%) and increased cardiovascular risk (3.8%). The primary justification for not prescribing thyroid hormone therapy was the normalization of TFTs (78.9%), followed by unknown reasons (20.5%), and other reasons (0.6%).

### 3.4. Determinants of Thyroid Hormone Replacement Therapy

We examined the factors associated with the prescription of LT4 therapy for SCH by performing a multivariable logistic regression analysis. White race (OR = 4.50, 95% CI 1.19 to 17.08, *p* = 0.026) and index TSH level as a continuous variable [OR = 1.71, 95% CI 1.24 to 2.35, *p* = 0.001; for every SD increase (1.6 mIU/L)] were associated with higher odds of treatment (Table 5). The initial LT4 dose for the treated patients was analyzed by fitting univariate linear regression models, considering age, weight, index TSH level, sex, race, cardiovascular disease, and atrial fibrillation as potential predictors. None of these predictors were individually statistically significant; thus, no predictors were selected for a multivariable model.

## 4. Discussion

In this retrospective cohort study, we found that approximately three in 10 Veterans with SCH were prescribed LT4 therapy. Only 49.2% of the patients in the treated group, compared to 97.0% of the patients in the untreated group, underwent confirmatory testing before LT4 therapy was initiated. We also found that testing for thyroid autoimmunity is rarely performed. In a multivariable analysis, index TSH level and White race were associated with higher odds of LT4 therapy initiation.

The prevalence of treated SCH in our study falls within the range documented in previous reports in the United States. In a cohort study of 450 patients living within 120 miles of Mayo Clinic (Rochester, MN, United States), LT4 therapy was prescribed for 39% of the patients with SCH defined as TSH 5.1–10.0 mIU/L with FT4 levels in the normal range from 1 January 1995 through 31 December 1996 [21]. Patients were randomly selected according to their thyroid autoimmunity status, which was positive in 43.8%, negative in 40.7%, and not checked in 15.5% of the patients [21]. This was significantly different in our study, where the vast majority of patients did not have thyroid autoimmunity labs, suggesting that clinicians in our institution do not utilize this test in their decision-making process for LT4 therapy initiation. In a multivariable analysis, the statistically significant determinants of LT4 therapy were TSH level, FT4 level, and thyroid peroxidase antibody status [21]. Recently, we conducted a 4-academic center (3 in the United States and 1 in Mexico) retrospective cohort study of 796 patients with SCH from 1 January 2016 through 31 December 2018 and found that 21% received LT4 treatment during the same time period as the present study [14]. In a multivariable logistic regression model, female sex and index TSH level were associated with higher odds of LT4 treatment [14]. In both studies [14,21], the majority of the included patients were female (69.3% [21] and 65.2% [14]), which differs from our study population (female 9%).

In our study, TSH level was a significant determinant for LT4 initiation, and the group of patients who were prescribed LT4 therapy had significantly higher TSH levels [median (IQR), 6.98 (2.06) mIU/L vs. 6.14 (1.10) mIU/L, *p* = 0.0002] compared to the untreated patients. Although the difference does not appear to be clinically significant, this finding is consistent with most guidelines recommending treatment for patients with SCH and higher TSH levels, particularly when TSH levels are above 7–10 mIU/L [4,10,22]. Indeed, we found that the LT4-treated group had more patients with TSH levels ≥ 10 mIU/L (11.1% vs. 3.0%, *p* = 0.021) compared to the untreated group.

Guidelines recommend confirming the diagnosis of SCH by repeating TFTs before initiating LT4 therapy [4,10]. This recommendation is based on the finding that most patients who are diagnosed with SCH have a TSH level between 4 and 10 mIU/L [7], and the majority of individuals with TSH level < 10 mIU/L normalize without any intervention [8]. In our study, for more than half of the treated patients, the decision to treat SCH was based on only one set of abnormal thyroid test results (unconfirmed SCH). When thyroid levels were retested, they normalized in 72% of the patients. This finding is consistent with those of previous studies [8,14] and adds to the evidence of LT4 overuse [15]. In a 3-academic center retrospective cohort study of 977 patients who were prescribed LT4 for the first time, it was found that in 45% of cases, the decision to initiate LT4 was based solely on a single TSH value (without concurrent thyroxine levels or previous abnormal TSH results) or non-confirmed mild SCH (TSH < 10 mIU/L) [15]. This practice contrasts with the current guidelines and underscores the need for targeted implementation strategies, particularly for primary care clinicians who are at the forefront of caring for these patients, to promote the evidence-based use of LT4 for SCH.

If we apply available prevalence data from the general population [3], up to two million Veterans may have been diagnosed with SCH and started on LT4 therapy inappropriately, leaving them at risk of preventable morbidity and burden. As the prevalence of SCH increases with age, there is an increased risk for overtreatment among nearly one-half of Veterans who are ≥65 years [23]. There is a prevailing perception among clinicians that LT4 is an easy pill to take, requires minimal monitoring, and is rarely associated with severe side effects [24]. However, among adults over 65 years who take LT4, ~50% had a TSH concentration < 0.45 mIU/L due to LT4 over-replacement [16,17]. Importantly, these patients can exhibit symptoms of hyperthyroidism (e.g., anxiety) and have an increased risk of arrhythmias, cardiovascular disease, bone loss, fractures, cognitive impairment, and dementia [16,18,20]. A cohort study assessing the relationship between thyroid hormone treatment intensity and cardiovascular mortality using data from the Veterans Health Administration on 705,307 adults who received thyroid hormone treatment showed that 10.8% died of cardiovascular causes and exogenous hyperthyroidism was associated with up to 47% increased risk of cardiovascular mortality compared with euthyroidism [19]. Critically, a recent randomized clinical trial also found a signal of harm, with more deaths in those treated for SCH [25]. In addition, taking LT4 requires changes in daily habits, e.g., administering 30–60 min prior to eating, monitoring of effects, and clinic and laboratory encounters [17]. LT4 administration often contributes to geriatric polypharmacy, leading to adverse effects, drug interactions, and increased healthcare utilization and expenditure [26]. This highlights the importance that clinicians at least confirm SCH before considering LT4 therapy as a strategy to reduce SCH overdiagnosis and subsequent overtreatment.

In our study, which included 87% White population and <1% Hispanics, we found that White race was a significant determinant for LT4 initiation in patients with SCH, suggesting disparities in the initiation of LT4. We had previously shown that in a large cohort of pregnant women in the United States, LT4 initiation for SCH was impacted by nonclinical factors such as patient race/ethnicity, with Hispanic women nearly 30% less likely and Asian women 47% more likely to start LT4 treatment compared with White women, suggesting disparities in health care access and quality [27]. In addition, in a cross-sectional study of a representative sample of adults with hypothyroidism in the United States, people of Hispanic ethnicity had lower odds of receiving adequate treatment for hypothyroidism compared to non-Hispanic White individuals [28]. However, in a study examining the distribution of non-evidence-based LT4 prescriptions, no differences were observed between racial groups [15]. More research is needed to specifically examine racial- and ethnic-related disparities in the context of hypothyroidism care and to understand the underlying causes.

To our knowledge, this is the first study to provide evidence for the prevalence of LT4-treated SCH in Veterans and the determinants of LT4 therapy initiation in this patient population. There were some limitations to our study. The study population includes Veterans receiving care at a Veterans Affairs medical center, which could introduce selection bias; whether our findings are generalizable to other patient populations (e.g., uninsured patients, patients with commercial health insurance, younger patients, etc.) is unclear. Despite including multiple confounders in our analysis, residual unmeasured confounding factors were possible. Moreover, the retrospective design of our study also presents certain inherent limitations. Using data from EHR may result in lower accuracy compared to data captured prospectively with a standardized survey from longitudinal cohorts. This is particularly true for information that depends heavily on the clinician’s effort and time spent documenting patient data (e.g., presence of hypothyroidism-related symptoms and thyroid exam findings). Additionally, missing or unclear data in the EHR, which required clarification that we were unable to obtain, were not included in the analyses.

In conclusion, we found that 27.5% of Veterans diagnosed with SCH were treated with LT4 therapy. Index TSH level and White race were significant determinants of LT4 treatment initiation for SCH. Confirmatory TFTs and thyroid autoimmunity testing were underused. Further research is required to develop effective implementation strategies that enhance the evidence-based and equitable use of LT4 in patients with SCH.

## Figures and Tables

**Table 1:** Demographic and social characteristics of the treatment groups.

Variable	All (N = 229)	Treated (N = 63)	Untreated (N = 166)	*p*-Value
Age (yrs.) *	68 (17)	67 (15)	68 (18)	0.72
Age ≥ 65 yrs	65.5% (150)	69.8% (44)	63.9% (106)	0.44
Sex				0.46
Female	10.0% (23)	12.7% (8)	9.0% (15)	
Male	90.0% (206)	87.3% (55)	91.0% (151)	
Race				0.039
White	87.2% (198/227)	95.2% (59/62)	84.2% (139/165)	
Black	11.5% (26/227)	3.2% (2/62)	14.5% (24/165)	
Other	1.3% (3/227)	1.6% (1/62)	1.2% (2/165)	
Unknown	0.9% (2)	1.6% (1)	0.6% (1)	
Ethnicity				0.47
Hispanic	0.9% (2/224)	1.6% (1/61)	0.6% (1/163)	
Non-Hispanic	99.1% (222/224)	98.4% (60/61)	99.4% (162/163)	
Unknown	2.2% (5)	3.2% (2)	1.8% (3)	
Marital status				0.87
Married	59.4% (136)	57.1% (36)	60.2% (100)	
Divorced	26.6% (61)	30.2% (19)	25.3% (42)	
Single	7.9% (18)	6.3% (4)	8.4% (14)	
Widowed	6.1% (14)	6.3% (4)	6.0% (10)	
Employment status				0.85
Employed	27.6% (63/228)	28.6% (18)	27.3% (45/165)	
Unemployed	42.5% (97/228)	44.4% (28)	41.8% (69/165)	
Retired	29.8% (68/228)	27.0% (17)	30.9% (51/165)	
Unknown	0.4% (1)	0.0% (0)	0.6% (1)	

* Data are presented as median (interquartile range).

**Table 2:** Reported hypothyroidism-related symptoms per treatment group.

Symptom	All (N = 229)	Treated (N = 63)	Untreated (N = 166)	*p*-Value
Weight gain	4.8% (11)	7.9% (5)	3.6% (6)	0.18
Fatigue	7.9% (18)	9.5% (6)	7.2% (12)	0.59
Headaches	8.7% (20)	9.5% (6)	8.4% (14)	0.80
Muscle weakness	4.4% (10)	7.9% (5)	3.0% (5)	0.14
Edema	3.1% (7)	3.2% (2)	3.0% (5)	>0.99
Depression	6.6% (15)	9.5% (6)	5.4% (9)	0.37
Constipation	4.4% (10)	6.3% (4)	3.6% (6)	0.47
Brittle nails	0.4% (1)	0.0% (0)	0.6% (1)	>0.99
Menstrual concerns	4.4% (1/23)	12.5% (1/8)	0.0% (0/15)	0.35
Neck pain	5.7% (13)	4.8% (3)	6.0% (10)	>0.99
Memory concerns	6.1% (14)	7.9% (5)	5.4% (9)	0.54
Impaired cognition	6.1% (14)	6.3% (4)	6.0% (10)	>0.99
Other symptoms	10.9% (25)	7.9% (5)	12.0% (20)	0.48
Asymptomatic	27.1% (62)	20.6% (13)	29.5% (49)	0.19

**Table 3:** Comorbidities per treatment group.

Comorbidity	All (N = 229)	Treated (N = 63)	Untreated (N = 166)	*p*-Value
Heart failure	7.4% (17)	4.8% (3)	8.4% (14)	0.41
Atrial fibrillation	12.7% (29)	14.3% (9)	12.0% (20)	0.66
Thyroid nodule(s)	1.7% (4)	3.2% (2)	1.2% (2)	0.30
Goiter	0.4% (1)	0.0% (0)	0.6% (1)	>0.99
Osteoporosis	4.8% (11)	4.8% (3)	4.8% (8)	>0.99
Hyperlipidemia	61.6% (141)	69.8% (44)	58.4% (97)	0.13
Myocardial infarction	19.7% (45)	22.2% (14)	18.7% (31)	0.58
Depression	30.1% (69)	31.7% (20)	29.5% (49)	0.75
PTSD	14.4% (33)	19.0% (12)	12.7% (21)	0.29
Other psychological conditions	15.3% (35)	11.1% (7)	16.9% (28)	0.31
Stroke	5.7% (13)	3.2% (2)	6.6% (11)	0.52
Dementia	2.6% (6)	1.6% (1)	3.0% (5)	>0.99
Hypertension	68.1% (156)	69.8% (44)	67.5% (112)	0.75
Myopathies	1.3% (3)	0.0% (0)	1.8% (3)	0.56
PVD	1.3% (3)	1.6% (1)	1.2% (2)	>0.99
COPD	8.3% (19)	7.9% (5)	8.4% (14)	>0.99
Connective tissue disorders	4.4% (10)	1.6% (1)	5.4% (9)	0.29
Liver disease	0.9% (2)	0.0% (0)	1.2% (2)	>0.99
Diabetes mellitus	32.3% (74)	36.5% (23)	30.7% (51)	0.43
Leukemia	0.4% (1)	0.0% (0)	0.6% (1)	>0.99
Lymphoma	0.9% (2)	0.0% (0)	1.2% (2)	>0.99
Cardiovascular disease				0.96
Has disease	25.8% (59)	25.4% (16)	25.9% (43)	
At risk of disease	69.9% (160)	71.4% (45)	69.3% (115)	
Not at risk	4.4% (10)	3.2% (2)	4.8% (8)	
No comorbidities	3.1% (7)	1.6% (1)	3.6% (6)	0.68

PTSD, post-traumatic stress disorder; PVD, peripheral vascular disease; COPD, chronic obstructive pulmonary disease.

**Table 4:** Clinical and biochemical characteristics of each treatment group.

Variable	All (N = 229)	Treated (N = 63)	Untreated (N = 166)	*p*-Value
Weight (kg) *	91.1 (25.5)	91.1 (24.3)	90.9 (25.6)	0.82
Body mass index (kg/m^2^) *	29.00 (7.55)	29.77 (6.67)	28.86 (7.73)	0.25
Systolic BP (mm Hg)	133.2 (17.2)	134.3 (18.5)	132.8 (16.8)	0.58
Diastolic BP (mm Hg)	77.1 (11.8)	77.2 (12.1)	77.0 (11.6)	0.92
Heart rate (bpm) *	73 (19)	75 (21)	72 (19)	0.42
Current smoker	31.7% (64/202)	34.0% (18/53)	30.9% (46/149)	0.73
Missing	11.8% (27)	15.9% (10)	10.2% (17)	
Family history of thyroid disease	42.9% (9/21)	60.0% (3/5)	37.5% (6/16)	0.61
Missing	90.8% (208)	92.1% (58)	90.4% (150)	
Abnormal thyroid exam	0.8% (1/128)	2.9% (1/35)	0.0% (0/93)	0.27
Missing	44.1% (101)	44.4% (28)	44.0% (73)	
FT4 level (ng/dL)	0.81 (0.13)	0.79 (0.15)	0.82 (0.12)	0.23
Index TSH (mIU/L) *	6.33 (1.48)	6.98 (2.06)	6.14 (1.10)	0.0002
Index TSH ≥ 10 mIU/L	5.2% (12)	11.1% (7)	3.0% (5)	0.021
Confirmation TSH performed	83.8% (192)	49.2% (31)	97.0% (161)	<0.0001
Confirmation TSH abnormal	28.1% (54/192)	96.8% (30/31)	14.9% (24/161)	<0.0001
TPOAb/TgAb assessed	1.3% (3)	3.2% (2)	0.6% (1)	0.18
TPOAb/TgAb positivity	66.7% (2/3)	50.0% (1/2)	100% (1/1)	>0.99

* Data are presented as median (interquartile range). BP, blood pressure; FT4, free thyroxine; TSH, thyrotropin; TPOAb, thyroid peroxidase antibody; TgAb, thyroglobulin antibody.

**Table 5:** Multivariable logistic regression model examining covariates associated with levothyroxine treatment.

Term	Referent	Odds Ratio	95% CI	*p*-Value
Age (yrs.)	Δ = 13.6 yrs. *	1.14	0.83 to 1.58	0.41
Female	Male	1.45	0.51 to 4.16	0.49
White race	Not White	4.50	1.19 to 17.08	0.026
Any thyroid symptom	No symptoms	1.61	0.77 to 3.38	0.20
Index TSH (mIU/L)	Δ = 1.6 mIU/L *	1.71	1.24 to 2.35	0.001

* The referent changes (Δ) for the continuous variables in the model represent a one standard deviation change; TSH, thyrotropin.

## Data Availability

The data sets used and/or analyzed during the current study are available from the corresponding author upon reasonable request.
